# Effectiveness of applying auricular acupressure to treat insomnia: a systematic review and meta-analysis

**DOI:** 10.3389/frsle.2024.1323967

**Published:** 2024-04-11

**Authors:** Li Jun, Li Xiong, Yu Wen, Wang Yongxiang

**Affiliations:** ^1^Zigong First People's Hospital, Zigong, Sichuan, China; ^2^Second Xiangya Hospital, Central South University, Changsha, Hunan, China

**Keywords:** auricular point, insomnia, meta-analysis, systematic review, estazolam

## Abstract

**Background:**

Insomnia affects the quality of life of a significant number of individuals worldwide. Despite the fact that pharmaceutical sleep treatments have shown brief enhancements in sleep quality, these are still not recommended for the long-term management of sleep issues. To deal with this problem, our study aims to assess the effectiveness of auricular acupressure for treating insomnia by conducting a systematic review and meta-analysis.

**Method:**

Data from randomized controlled trials (RCTs) of auricular acupressure for insomnia was collected from five English-language databases (Cochrane Central Register of Controlled Trials, MEDLINE, EMBASE, AMED, and CINAHL) and four Chinese databases (CBM, CNKI, CQVIP, and Wanfang). Relevant data were extracted by two reviewers. *I*^2^ statistics were adopted to appraise heterogeneity. A network meta-analysis was applied to compare the effect of auricular acupressure with other methods.

**Result:**

In all, 23 RCTs involving a total of 1,689 patients were included. The results demonstrated a significant decrease in the Pittsburgh Sleep Quality Index (PSQI) score for the intervention group compared to the control group [SMD = −1.30, 95% CI (−1.65, −0.96), *I*^2^ = 90%]. Furthermore, the group receiving auricular acupressure in addition to usual care showed a lower PSQI score compared to the usual care group [SMD = −1.13, 95% CI (−1.33, −0.93), *I*^2^ = 23%]. Auricular acupressure was found to enhance the effectiveness of estazolam in improving PSQI score, with the combination of auricular acupressure and estazolam resulting in a lower PSQI score [MD = −4.8, 95% CI (−7.4, −2.1)]. Importantly, no serious adverse events were reported. In patients with insomnia following stroke, the intervention group (which received auricular acupressure) exhibited a lower PSQI score compared to the control group [SMD = −0.74, 95% CI (−1.03, −0.46), *I*^2^ = 0%]. Similarly, in patients with insomnia related to cancer, the intervention group (receiving auricular acupressure) demonstrated a lower PSQI score compared to the control group [SMD = −0.99, 95% CI (−1.37, −0.61), *I*^2^ = 0%].

**Conclusion:**

The effects of auricular acupressure on insomnia are comparable to those of estazolam. Furthermore, auricular acupressure can serve as a complementary treatment to estazolam or other interventions, effectively improving symptoms of insomnia.

## Introduction

Insomnia is characterized by an enduring challenge in initiating or maintaining sleep, as well as waking up earlier than desired. This sleep disorder has emerged as the second-most-prevalent mental disorder, with approximately one third of adults in the general population experiencing at least one symptom of insomnia. Moreover, ~10% of individuals satisfy the diagnostic criteria for insomnia (Morin et al., 2006a; Ito and Inoue, 2015). Insomnia is associated with a range of diseases, encompassing both mental and physical conditions. This sleep disorder exerts a detrimental effect on daytime cognitive functions, including alertness, memory, attention, and executive function (Zhao et al., 2018; Wardle-Pinkston et al., 2019). A poorer quality of sleep has been found to be associated with increased aggression and impaired global cognitive and memory performance in subsequent assessments (Van Veen et al., 2021; Baril et al., 2022). Insomnia has been associated with hypertension and type 2 diabetes, thereby contributing to an increased cardiovascular risk (Johnson et al., 2021; Maiolino et al., 2021). Insomnia has been found to be associated with increased risks of developing atrial fibrillation and heart failure, according to multiple studies (Li et al., 2021; Mahmood et al., 2021).

The high incidence and debilitating nature of insomnia contribute to significant health and economic challenges, including substantial medical expenses and indirect costs resulting from work disruptions and delays (Léger and Bayon, 2010). Based on conservative estimates, the annual economic burden of insomnia is estimated to range from US $92.5 billion to $107.5 billion (Stoller, 1994). Since its emergence in 2019, the COVID-19 pandemic has affected a considerable population globally. Furthermore, individuals who have recovered from the disease may experience various psychological problems, including insomnia, as a result of the factors associated with the novel coronavirus infection (Pappa et al., 2020). Insomnia should be a public health concern. Pharmacological interventions for sleep provide temporary improvements in sleep but are not recommended for addressing chronic sleep problems due to limited effectiveness and the risk of dependence and other potential adverse effects (National Institute for Health Care and Excellence, 2004; Espie, 2009). Non-pharmacological interventions have demonstrated effectiveness in improving sleep, both in individuals with primary insomnia (Edinger and Means, 2005; Morin et al., 2006b) and in those with sleep problems associated with medical and psychiatric disorders (Smith et al., 2005; Riemann and Perlis, 2009; Geiger-Brown et al., 2015).

Auricular acupressure involves applying pressure using small objects, ~2 mm in diameter (e.g., botanical seeds or magnetic metal pellets), to specific acupoints on the patient's ear using small pieces of waterproof tape to mimic the effects of acupuncture (Yeh et al., 2013). As a non-invasive method, auricular acupressure draws on auricular acupuncture and other needle-based acupuncture therapies in its development, providing a safer alternative with reduced risk of infection. Furthermore, patches used for auricular acupressure have the capacity to remain in place for up to 1 month, potentially extending the therapeutic period without requiring constant and direct provider oversight and thus reducing the number of necessary visits to a healthcare practitioner and subsequent medical costs (McDonough et al., 2008). Moreover, the daily routines of patients undergoing auricular acupressure are minimally disturbed due to the convenience of self-administering the technique (Phang et al., 2021).

Discovering a simple, safe, effective, and affordable intervention for insomnia is crucial in the context of global health. Auricular acupressure fulfills these criteria. This study aimed to conduct a structured systematic review and meta-analysis to assess the effectiveness and safety of auricular acupressure for insomnia.

## Methods

### Search strategy

Our team conducted a comprehensive systematic search to identify relevant published articles. The search encompassed five English-language databases (Cochrane Central Register of Controlled Trials, MEDLINE, EMBASE, AMED, and CINAHL) and four Chinese databases (CBM, CNKI, CQVIP, and Wanfang), and five clinical trial registration databases (ClinicalTrials.gov, ANZCTR, EU-CTR, ChiCTR, and ICTRP) from their inception until July 2023. The retrieval strategy employed the following keywords: ([auricular point pressing] OR [auricular acupressure]) AND ([insomnia] OR [sleep disorders]).

### Selection criteria

Studies meeting the following criteria were included in our study: (1) they were clinical trials, (2) the trial intervention involved the use of auricular point acupressure specifically designed to improve sleep quality, (3) the trial included a control condition (sham or drugs, among others), (4) the full text of the study was available, (5) the articles were published in English or Chinese, and (6) the main results were assessed using the Pittsburgh Sleep Quality Index (PSQI). Studies were excluded if they met any of the following criteria: (1) they focused on insomnia as a secondary symptom; (2) the trial intervention was designed to treat parasomnias, sleep apnea, or fatigue, as these conditions have distinct causal factors from other sleep problems; and (3) the full text of the study was not available.

### Study selection

Duplicates were removed through electronic and manual means. Two reviewers (YXW and LJ) independently reviewed the title, abstract, and full text of each article. Any disagreements about the title or content of the summary were deferred to the subsequent stage of the process. To guarantee the inclusion of all potentially eligible articles, a random sample of 10% of articles was independently reviewed by a third reviewer (LX). The full texts of all potentially relevant articles were obtained and independently assessed for eligibility against the inclusion/exclusion criteria by two reviewers (YXW and LJ). Any disagreements regarding eligibility were resolved through discussions between two reviewers (LX and YW), and if consensus was not reached, a third reviewer (XYW) was consulted.

### Data extraction

Data collection and extraction adhered to the guidelines set forth by the Preferred Reporting Items for Systematic Review and Meta-Analyses (PRISMA). Two reviewers (YXW and LJ) conducted a comprehensive search, independently reviewed all relevant studies, and selected those that met the inclusion criteria. Any disagreements regarding eligibility or data extraction were resolved through discussions between the two reviewers. In cases in which an agreement could not be reached, the final decision was made by other team members (LX).

### Data analysis

All eligible studies were included in a random-effects meta-analysis. Meta packages (Balduzzi et al., 2019) were used to conduct the meta-analysis. For each study, standardized mean differences of PSQI at post-intervention and their corresponding 95% confidence intervals were computed, and the weights were determined based on the sample size using the random-effects model. To assess statistical heterogeneity, the χ^2^ and *I*^2^ statistics were employed. Sensitivity analyses were performed on the data from the studies, and subgroup analyses were conducted. Publication bias was examined by visually inspecting a funnel plot. Moreover, the gemtc package was used to compare the effects of different interventions across multiple research studies.

The risk of bias and the quality of the observational studies were evaluated using the Cochrane Risk of Bias tool. We analyzed the auricular points for insomnia in the included studies and calculated the frequency of occurrence for each factor. Additionally, we constructed a network to illustrate the relationship between auricular points and disease.

## Results

### Study selection

**Figure 1:**
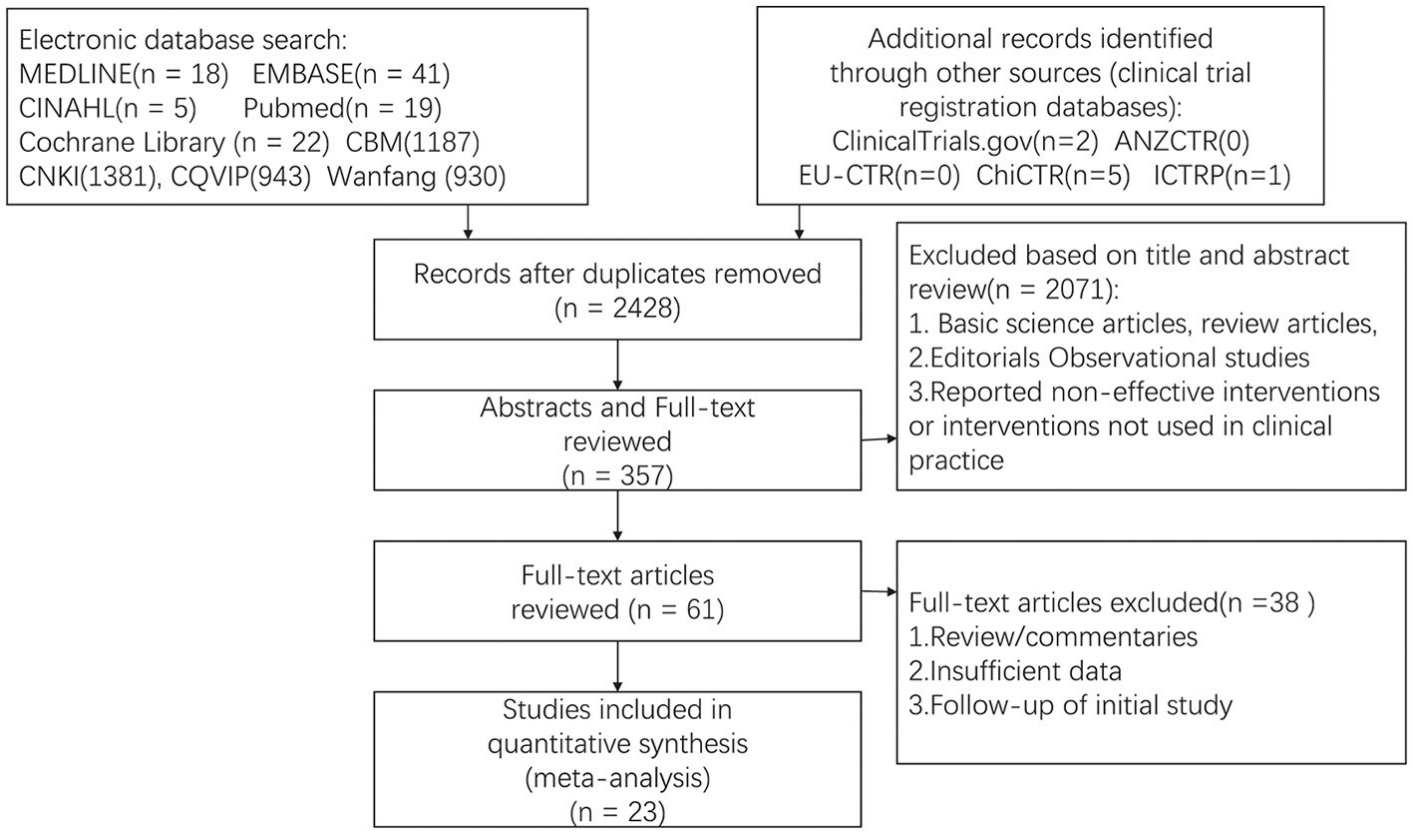
Preferred reporting items for systematic review and meta-analyses flow diagram.

A total of 23 studies (*n* = 1,689 patients) were included in this meta-analysis (Lin, 2007; Hu Wei et al., 2010; Liyue, 2012; Lihua, 2013; Wu Xiuqing et al., 2013; Zhou Min and Xiuqing, 2013; Rui, 2014; Yang Guigui and Yali, 2014; Juhong, 2015; Yin Chunyue and Yong, 2015; Wang Bo et al., 2016; Lin Nijing and Yanping, 2018; Liu Liuyan and Luping, 2018; Lu, 2018; Fan Wei et al., 2019; Li Aiyun, 2019; Pingting, 2019; Xiuping, 2019; Zhong Yuanchun and Xuan, 2019; Wang Zihao, 2020; Xinai, 2020; Yang, 2020; Limei, 2021) (details are provided in Supplementary Table 1). A PRISMA flow chart illustrating the search results is presented in Figure 1. All the studies included in this analysis were conducted in China, comprising 16 randomized controlled trials and seven controlled clinical trials. Specifically, four clinical trials focused on uremia patients with insomnia, three clinical trials that targeted stroke patients with insomnia, six clinical trials that examined hypertension patients with insomnia, and four clinical trials that investigated patients with primary insomnia. The details of Patients, Interventions, Comparisons, and Outcomes for each article are presented in Table 1.

**Table 1:** The characteristics of each study.

**References**	**Participants (P)**	**Interventions (I)**	**Comparison (C)**	**Outcomes (O)**
Zhou Min and Xiuqing (2013)	Insomnia patient with uremia	Auricular acupressure	Lunesta	PSQI scores
Zhong Yuanchun and Xuan (2019)	Insomnia patient with uremia	Auricular acupressure	Usual care	PSQI scores
Yin Chunyue and Yong (2015)	Insomnia patient with stroke	Auricular acupressure + usual care	Usual care	PSQI scores
Yang Guigui and Yali (2014)	Primary insomnia patient	Auricular acupressure + estazolam	Estazolam	PSQI scores
Yang (2020)	Insomnia patient with cancer	Auricular acupressure + usual care	Usual care	PSQI scores
Xiuping (2019)	Insomnia patient with hypertension	Auricular acupressure + usual care	Usual care	PSQI scores
Xinai (2020)	Insomnia patient with coronary (heart) disease	Auricular acupressure + usual care	Usual care	PSQI scores
Wu Xiuqing et al. (2013)	Insomnia patient with uremia	Auricular acupressure	Usual care	PSQI scores
Wang Zihao (2020)	Insomnia patient with stroke	Auricular acupressure	Diazepam	PSQI scores
Wang Bo et al. (2016)	Insomnia patient with hypertension	Auricular acupressure + estazolam	Estazolam	PSQI scores
Rui (2014)	Insomnia patient with depression	Auricular acupressure + deanxit	Deanxit	PSQI scores
Pingting (2019)	Insomnia patient with cancer	Auricular acupressure + usual care	Usual care	PSQI scores
Lu (2018)	Insomnia patient with AIDS	Auricular acupressure	Sham	PSQI scores
Liyue (2012)	Insomnia patient with diabetes	Auricular acupressure	Usual care	PSQI scores
Liu Liuyan and Luping (2018)	Insomnia patient with hypertension	Auricular acupressure + usual care	Usual care	PSQI scores
Lin Nijing and Yanping (2018)	Insomnia patient with hypertension	Auricular acupressure + usual care	Usual care	PSQI scores
Lin (2007)	Primary insomnia patient	Auricular acupressure	Sham	PSQI scores
Limei (2021)	Insomnia patient with hypertension	Auricular acupressure + anti-hypertensive	Anti-hypertensive	PSQI scores
Lihua (2013)	Insomnia patient with stroke	Auricular acupressure	Estazolam	PSQI scores
Li Aiyun (2019)	Insomnia patient with hypertension	Auricular acupressure + usual care	Usual care	PSQI scores
Juhong (2015)	Primary insomnia patient	Auricular acupressure	Body acupoints	PSQI scores
Hu Wei et al. (2010)	Primary insomnia patient	Auricular acupressure	Diazepam	PSQI scores
Fan Wei et al. (2019)	Insomnia with uremia patient	Auricular acupressure	Estazolam	PSQI scores

PSQI, Pittsburgh Sleep Quality Index.

### Efficacy of auricular acupressure

**Figure 2:**
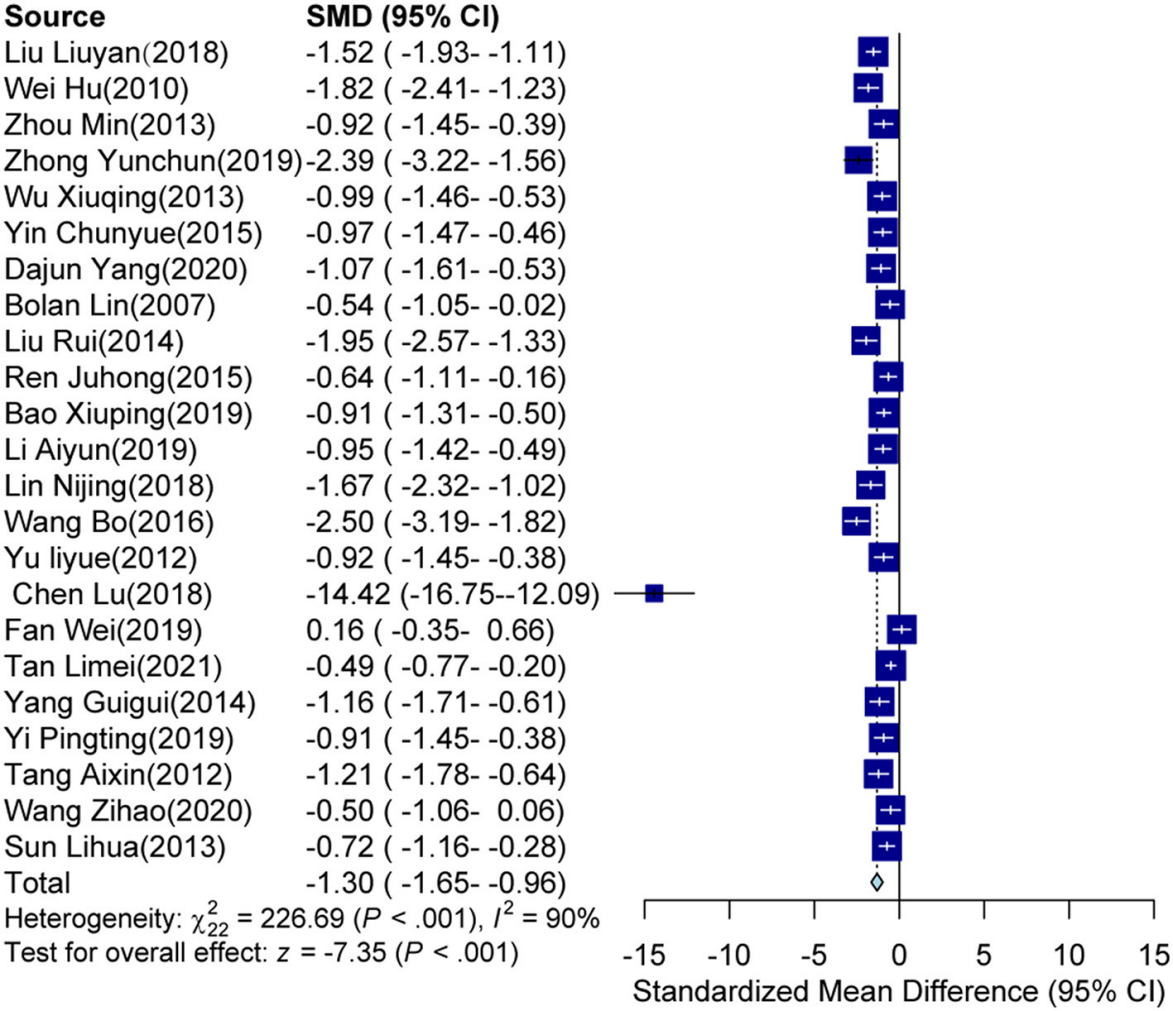
Forest plot for data included in the meta-analysis.

The pooled meta-analyses revealed that the intervention groups, which comprised either a single-use auricular acupressure or an associated control intervention, significantly decreased the PSQI score compared to the control groups[SMD = −1.30, 95% CI (−1.65, −0.96), *p* < 0.001; see Figure 2]. Sensitivity analyses indicated that the study conducted by Lu (2018) was a clear source of heterogeneity (see Supplementary Figure S1). Consequently, due to the high heterogeneity observed (*I*^2^ = 90%, *p* < 0.001), subgroup analyses were conducted to explore potential sources.

### Subgroup analyses

**Figure 3:**
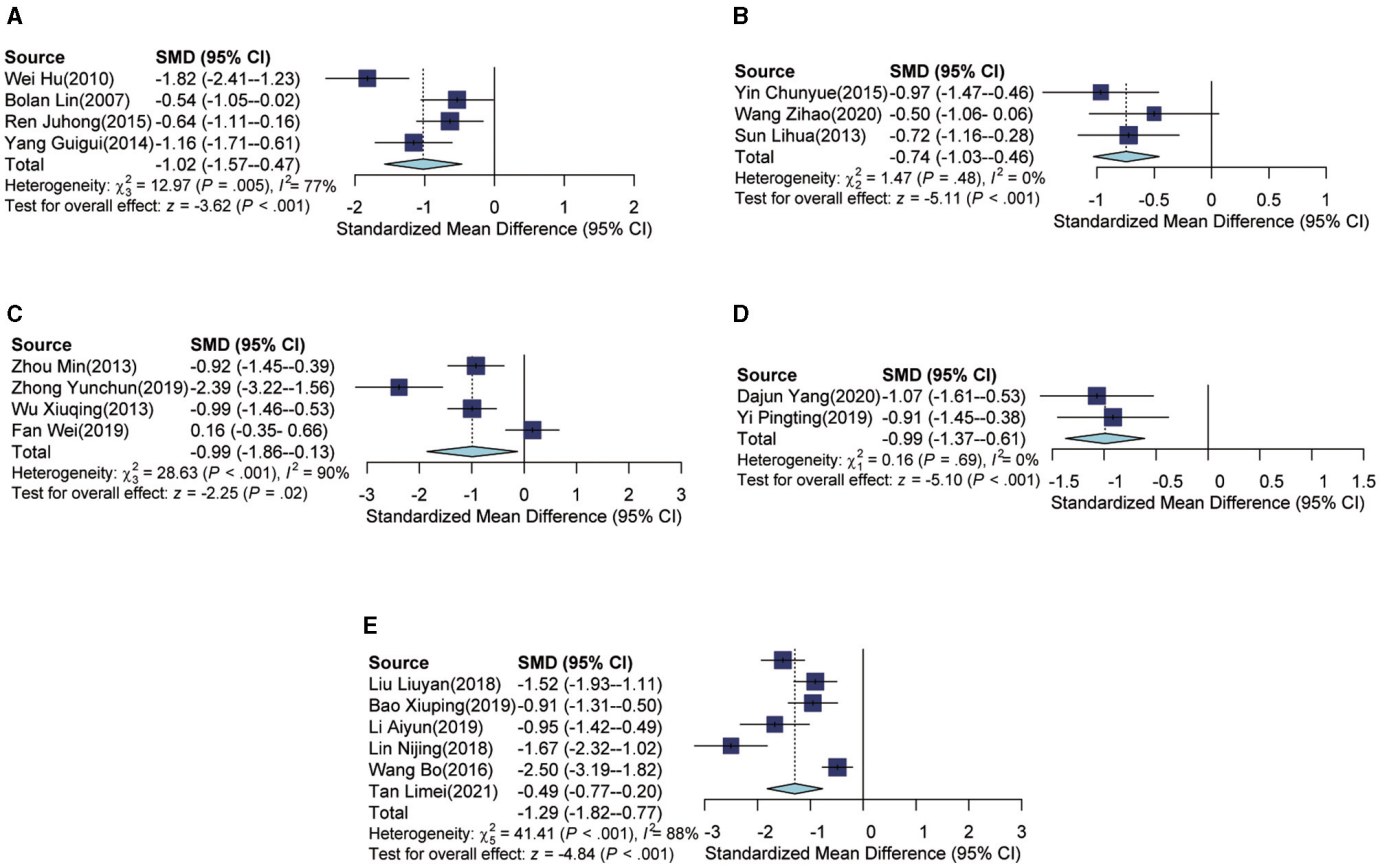
Forest plot according to subgroups of diseases. **(A)** The forest plot of primary insomnia. **(B)** The forest plot of insomnia patients with stroke. **(C)** The forest plot of insomnia patients with uremia. **(D)** The forest plot of insomnia patients with cancer. **(E)** The forest plot of insomnia patients with hypertension.

Based on the subgroup analysis of different diseases, it was found that in the four clinical trials involving patients with primary insomnia, the intervention group (which received auricular acupressure) exhibited significantly lower PSQI scores compared to the control group [SMD = −1.02, 95% CI (−1.57, −0.47), *p* < 0.001], with an *I*^2^ of 77% (see Figure 3A). Similarly, in the three clinical trials focusing on insomnia patients with stroke, the intervention group (also receiving auricular acupressure) had lower PSQI scores compared to the control group [SMD = −0.74, 95% CI (−1.03, −0.46), *p* < 0.001], and an *I*^2^ of 0% was observed (see Figure 3B). For the four clinical trials addressing insomnia patients with uremia, the intervention group (receiving auricular acupressure) had lower PSQI scores compared to the control group [SMD = −0.99, 95% CI (−1.86, −0.13), *p* < 0.001], with an *I*^2^ of 90% (see Figure 3C). In the two clinical trials involving insomnia patients with cancer, the intervention group (with auricular acupressure) had lower PSQI scores compared to the control group [SMD = −0.99, 95% CI (−1.37, −0.61), *p* < 0.001], and an *I*^2^ of 0% was obtained (see Figure 3D). Finally, in the six clinical trials examining insomnia patients with hypertension, the intervention group (including auricular acupressure) was associated with lower PSQI scores compared to the control group [SMD = −1.29, 95% CI (−1.82, −0.77), *p* < 0.001], and an *I*^2^ of 88% was observed (see Figure 3E).

**Figure 4:**
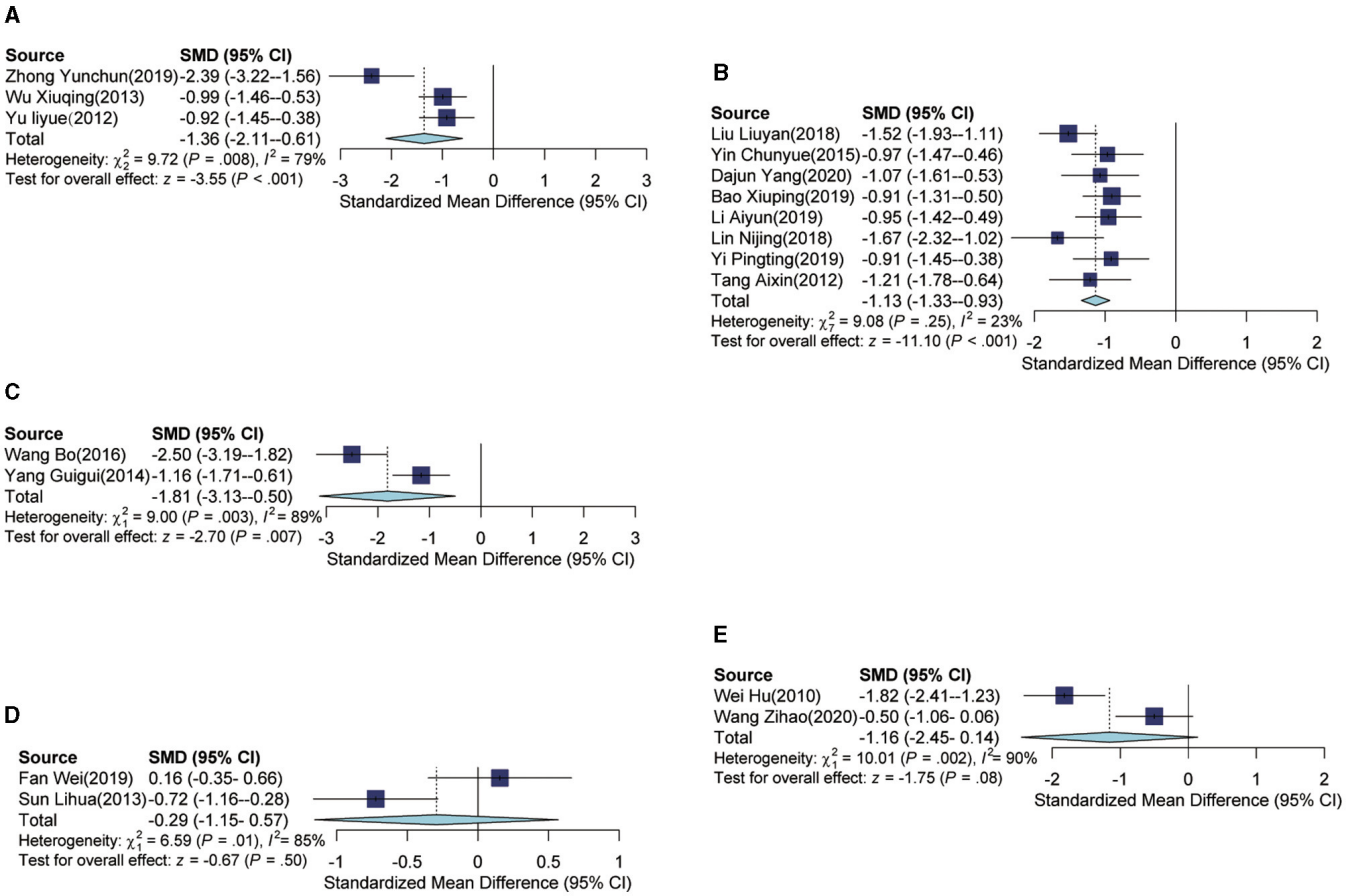
Forest plot according to subgroups of intervention. **(A)** Forest plot of auricular acupressure group vs. usual care group. **(B)** Forest plot of auricular acupressure plus usual care group vs. usual care group. **(C)** Forest plot of auricular acupressure plus estazolam group vs. estazolam group. **(D)** Forest plot of auricular acupressure group vs. estazolam group. **(E)** Forest plot of auricular acupressure vs. diazepam group.

In the analysis of different intervention subgroups, the auricular acupressure group demonstrated a significant association with lower PSQI scores compared to the usual care group [SMD = −1.36, 95% CI (−2.11, −0.61), *p* < 0.001], with an *I*^2^ of 79% (Figure 4A). When auricular acupressure was combined with usual care, there was also a significant decrease in PSQI scores compared to the usual care group [SMD = −1.13, 95% CI (−1.33, −0.93), *p* < 0.001], with an *I*^2^ of 23% (Figure 4B). Furthermore, the combination of auricular acupressure with estazolam resulted in lower PSQI scores when compared to the estazolam group [SMD = −1.81, 95% CI (−3.13, −0.50), *p* = 0.007], and an *I*^2^ of 89% was observed (Figure 4C). In addition, the auricular acupressure group exhibited lower PSQI scores when compared to the estazolam group [SMD = −0.29, 95% CI (−1.15, 0.57), *p* = 0.5), with an *I*^2^ of 85% (Figure 4D). Finally, lower PSQI scores were observed in the auricular acupressure group compared to the diazepam group, although the difference was not statistically significant [SMD = −1.16, 95% CI (−2.45, 0.14), *p* = 0.08], with an *I*^2^ of 90% (Figure 4E).

### Risk-of-bias assessment

**Figure 5:**
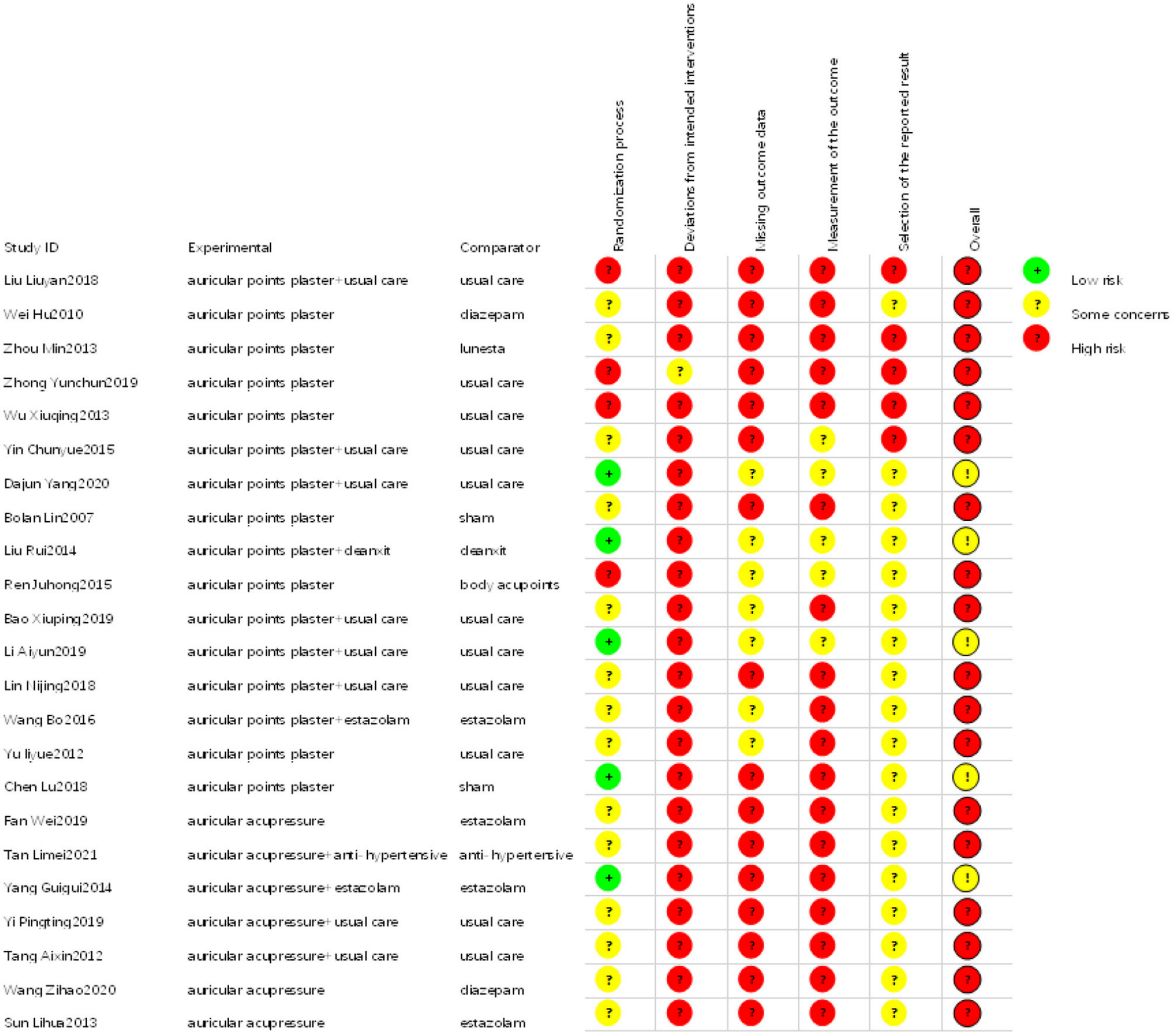
Risk-of-bias summary: review authors' judgments about each risk-of-bias item for each included study.

**Figure 6:**
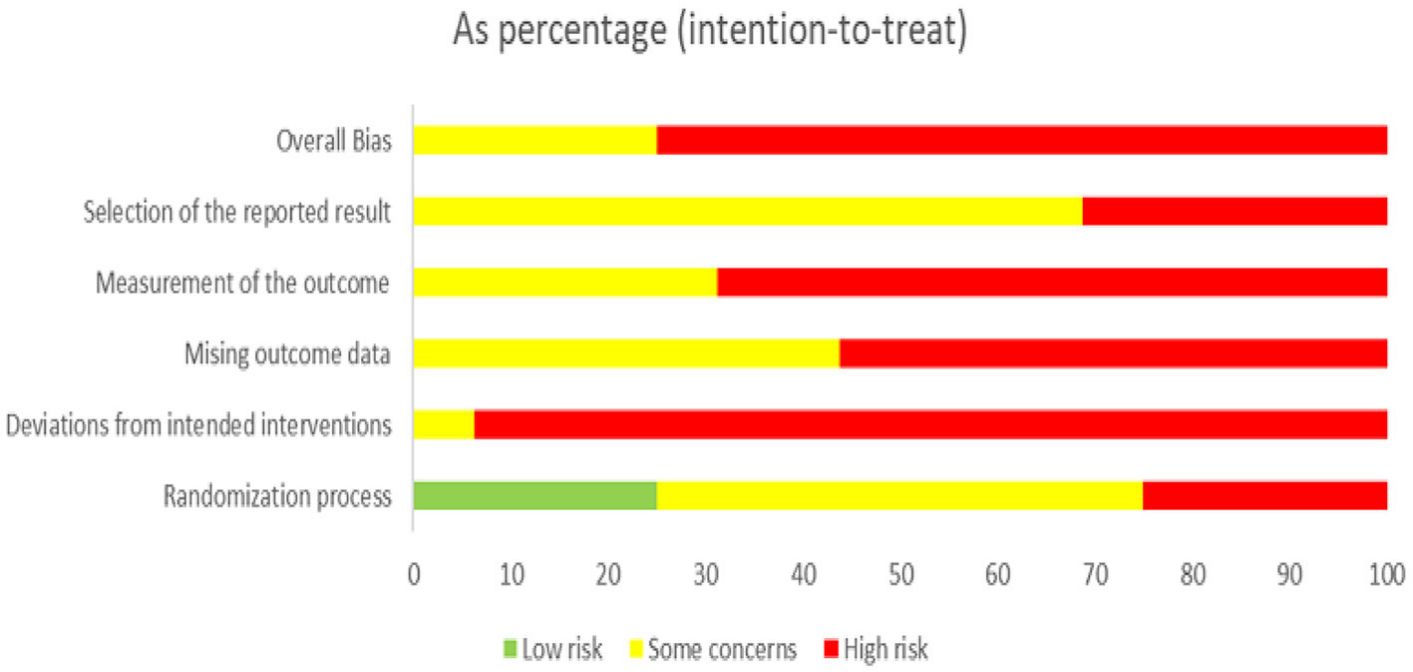
Risk-of-bias graph: review authors' judgments about each risk-of-bias item presented as percentages across all included studies.

Publication bias and quality assessments were analyzed in this study. Each study underwent a thorough quality assessment, which concluded that they were of high quality and demonstrated significant findings (Figures 5, 6). Publication bias was assessed through visual inspection of the funnel plot (Supplementary Figure S2) and the use of Egger regression analysis (*p*-value = 0.003994).

### Network meta-analysis

**Figure 7:**
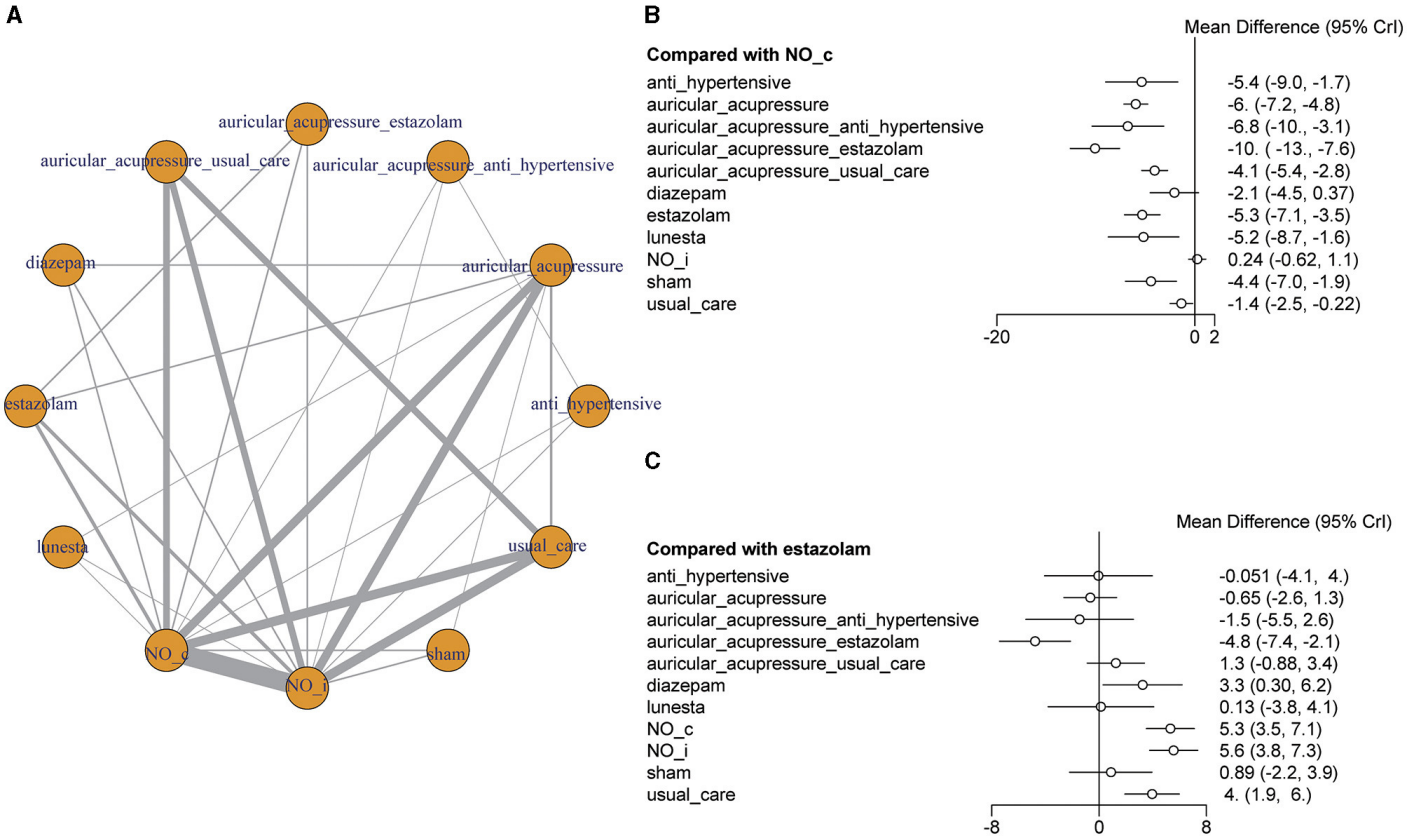
Network meta-analysis. **(A)** Network diagram. The nodes in the mesh represent different interventions, and the nodes and connections are weighted by the number of studies that contain direct comparison interventions. The bigger the node is, the more times it appears in the direct comparison, and the thicker the line is, the more the number of the direct comparison is. **(B)** The forest plot of different interventions vs. no intervention. **(C)** The forest plot of different interventions vs. estazolam.

The network geometries showed the treatment effects of all insomnia therapies from the included research (Figure 7A). Network meta-analysis revealed that auricular point plasters could reduce PSQI scores and work in coordination with other therapies (Figure 7B). Auricular acupressure could decrease the mean PSQI score [MD = −6, 95% CI (−7.2, −4.8)]. Usual care could decrease the mean PSQI score [MD = −1.4, 95% CI (−2.5, −0.22)], and auricular acupressure plus usual care could decrease the mean PSQI score [MD = −4.1, 95% CI (−5.4, −2.8)]. Estazolam could decrease the mean PSQI score [MD = −5.3, 95% CI (−7.1, −3.5)], and auricular acupressure plus estazolam could decrease the mean PSQI score [MD = −10, 95% CI (−13, −7.6)]. An antihypertensive agent could decrease the mean PSQI score [MD = −5.4, 95% CI (−9.0, −1.7)], and auricular acupressure plus an antihypertensive agent could decrease the mean PSQI score [MD = −6.8, 95% CI (−10, −3.1)].

Lunesta, estazolam, and diazepam are three common medications used to treat insomnia. Lunesta (eszopiclone) can slow brain activity to help patients sleep. Estazolam and diazepam, belonging to the benzodiazepines, can enhance the activity of a neurotransmitter of gamma-aminobutyric acid in the brain to treat insomnia. Compared with estazolam, auricular acupressure [MD = −0.65, 95% CI (−2.6, 1.3)], auricular acupressure plus usual care [MD = 1.3 95% CI (−0.88, 3.4)], auricular acupressure plus antihypertensive [MD = −1.5, 95% CI (−5.5, 2.6)], and Lunesta [MD = 0.13, 95% CI (−3.8, 4.1)] made no difference in PSQI score. Auricular acupressure could increase the effect of estazolam on PSQI scores, and auricular acupressure plus estazolam had lower PSQI scores [MD = −4.8, 95% CI (−7.4, −2.1); Figure 7C].

### Regular pattern of auricular point for insomnia

**Figure 8:**
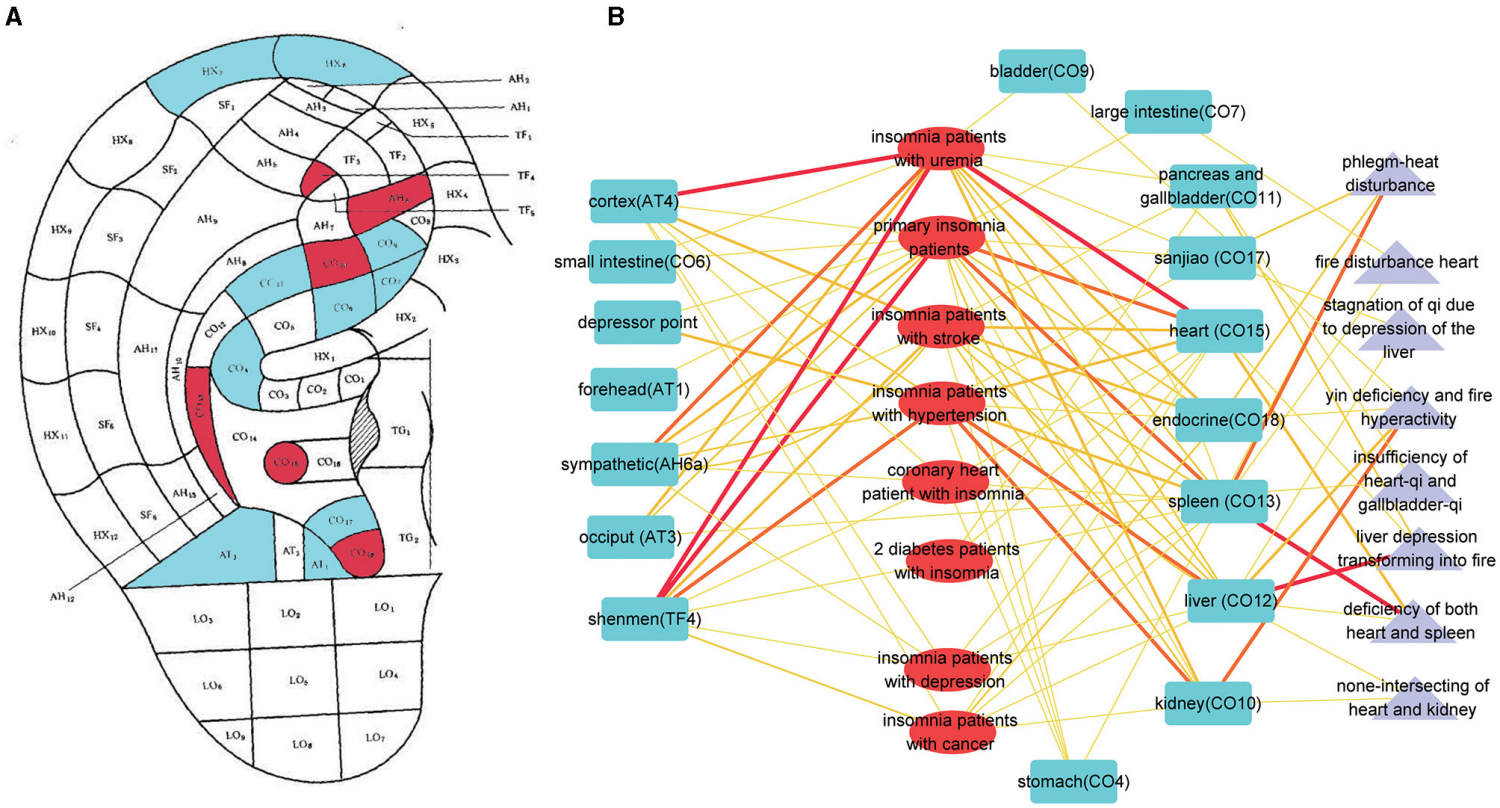
The pattern of auricular points for insomnia. **(A)** The auricular points' locations on the ear. Red means higher times to be adopted for insomnia. Blue means lower times to be adopted for insomnia. **(B)** The network of auricular points and different types of insomnia, including syndrome differentiation. Blue stands for auricular points. Red stands for the type of insomnia. Purple stands for dialectical type.

It was found that 18 points were used per China's auricular standards, and a frequency analysis indicated that seven of these points were used far more often than the others (details are presented in Supplementary Table 2): the liver (CO12), spleen (CO13), shenmen (TF4), heart (CO15), kidney (CO10), sympathetic (AH6a), cortex (AT4), and endocrine (CO18) points (Figure 8A). The network between diseases and auricular points is shown in Figure 8B (details are presented in Supplementary Table 3). In the network, there were eight dialectical types about insomnia (liver depression transforming into fire, phlegm-heat disturbance, yin deficiency and fire hyperactivity, deficiency of both heart and spleen, insufficiency of heart-qi and gallbladder-qi, none-intersecting of heart and kidney, syndrome of timidity due to deficiency of heart qi, fire disturbance heart).

## Discussion

This systematic review identified 23 studies that examined the effects of auricular acupressure in treating insomnia. Auricular acupressure, a traditional Chinese medicine therapy, has been practiced in China for more than 2,000 years and has gradually gained popularity in other countries over the past few centuries. The auricle, or outer ear, contains acupoints that can be stimulated through pressure. By applying pressure to these acupoints, the meridians can be stimulated. Each acupoint reflects the physiological and pathological condition of its corresponding body part and can be regulated through pressure stimulation to address any dysfunction (Kuo et al., 2016). Specifically, when a specific acupoint is pressurized, it triggers an effect accompanied by a warm, swelling pain sensation known as a qi sensation (Luo et al., 2021).

Insomnia often arises as a secondary dysfunction associated with various diseases. The studies investigated insomnia patients with both primary and secondary insomnia, including cases related to cancer, type 2 diabetes, depression, uremia, stroke, hypertension, and coronary heart disease. Consequently, insomnia can lead to symptoms like fatigue, restlessness, lethargy, and slow reactions, as well as headaches. Furthermore, these symptoms can contribute to the development of psychiatric disorders such as schizophrenia and depression, anxiety disorders, autonomic dysfunction, and other functional and systemic diseases, including those affecting the cardiovascular and digestive systems. In summary, insomnia becomes a perpetuating factor in a vicious cycle that negatively impacts overall health. Specific attention should be directed toward insomnia in patients with hypertension or cancer, considering the high number of hypertension patients and its association with stroke and coronary heart disease. In particular, quality of life holds significant importance for cancer patients, especially those in terminal stages. Insomnia plays a critical role in the development of hypertension and profoundly influences the quality of life for cancer patients.

In addition to that, the aging process in the brain often involves a significant decline in the microglial population and its associated functions, which can contribute to insomnia in older individuals (Choudhury et al., 2021). Moreover, the COVID-19 pandemic has profoundly impacted the health and wellbeing of millions of people worldwide, with an unprecedented scale and speed. COVID-19 is likely to have a substantial influence on psychological distress (Wang et al., 2002; Kumar and Nayar, 2021), which, in turn, has been linked to sleep disturbances. Thus, both physiological processes and the current public health environment contribute to the growing number of individuals suffering from insomnia. Given the inevitable trends of population aging and the ongoing COVID-19 pandemic, the prevalence of insomnia on a global scale is expected to reach unprecedented levels. Therefore, it is of utmost importance to prioritize efforts toward addressing this issue from a public health perspective.

In light of the outcomes and incidence trends associated with insomnia, the prevention and treatment of this condition can have significant benefits for overall health and the management of other diseases. While convenient, pharmaceutical drugs used for insomnia are often expensive and prone to side effects. Our research demonstrates that Lunesta, auricular acupressure, and estazolam can yield similar effects in treating insomnia. Additionally, when combined with estazolam, auricular acupressure can enhance the efficacy of its treatment. Although acupuncture and other physical therapies are cost-effective, they require professional skills, making self-administration by patients challenging. Auricular acupressure, by comparison, can be self-administered or performed with assistance and imposes minimal constraints in terms of time and space.

From a public health perspective, the most effective approach for combating insomnia is through public education on pressing ear acupoints. In cases in which insomnia is severe, auricular acupressure can complement drug interventions, reducing the reliance on sleeping pills. Promoting the use of auricular acupressure in the community can significantly reduce the human and financial resources dedicated to managing insomnia.

The PSQI is currently the most common tool for sleep assessment. Developed by Professor Buysse and his team at the University of Pittsburgh School of Medicine in 1989, it is used to evaluate the sleep quality and sleep disorders in adults, covering seven domains of sleep quality: sleep quality, sleep duration, sleep efficiency, sleep latency, sleep disturbances, medication use, and daytime dysfunction (Buysse et al., 1989). The PSQI can be used to assess individual sleep quality and sleep disorders, aiding health care professionals in diagnosing and monitoring the efficacy of sleep disorder treatments, as well as be utilized in scientific research. However, the PSQI has certain limitations as it is subject to subjective evaluation by the participants and potential memory biases, and it may not fully reflect the details or severity of specific sleep disorders. This aspect should be emphasized in clinical research on insomnia, and future studies need to develop more sensitive assessment tools corresponding to insomnia.

There were also some limitations encountered in this study. First, the quality of the included studies in this article was generally low. Out of the 23 studies, only five reported appropriate randomization methods, and none mentioned allocation concealment. This lack of robust methodology and transparency regarding the allocation process raises concerns about the reliability and validity of the findings. Second, the heterogeneity among the results of this study is pronounced. To understand the sources of heterogeneity, we conducted subgroup analyses based on comparing intervention methods and the types of diseases. It was found that these factors contributed partially to the observed heterogeneity. However, significant unexplained heterogeneity still persisted in the pooled results. One possible reason for this substantial unexplained heterogeneity could be the variations in acupoints and operational methods used across the studies. On one hand, shenmen and xin (Heart) acupoints are the acupoints specific to sleep, and almost all studies included these acupoints. However, there are some variations in the selection of additional acupoints among different studies for different types of patients. While these differences in acupoint selection may not alter the ultimate clinical efficacy, they could impact the strength of the treatment effect and contribute to heterogeneity. On the other hand, auricular acupressure requires the physical stimulation of acupoints, and there are significant individual differences among operators. It is challenging to ensure consistency in the intensity of acupressure and the sequence of pressing acupoints on the ear. These variations in manipulation could be a potential source of heterogeneity. Additionally, the number of trials investigating intervention methods and types of diseases varied, which could further contribute to the heterogeneity observed. Third, the studies included in this analysis were conducted exclusively in China, which limits the generalizability of the findings to other regions. These gaps in geographical representation and safety evidence highlight the need for further research and analysis in future studies.

This study found that in the clinical process of treating insomnia with auricular acupressure, there are certain differences in the auricular acupoints used by different researchers. Currently, the studies are all randomized controlled trials on auricular acupressure and non-auricular acupoint interventions, without comparative studies on the efficacy of different auricular acupoint protocols. It is necessary for future research to fill in the gaps in this area. This study also conducted statistical analysis on all studies on auricular acupressure, identifying high-frequency auricular acupoints and low-frequency auricular acupoints. However, whether there is a fundamental difference in the effects of these two types of auricular acupoints on insomnia, and the impact of different auricular acupoints on the process of insomnia (falling asleep, staying asleep), remains unsupported by research, which will be a focus of future studies.

In spite of these limitations, this systematic review has several strengths. First, it provides a comprehensive analysis of insomnia, with a focus on the underlying diseases in patients with insomnia. Second, it includes research published after 1989 and conducts a rigorous assessment of the quality of evidence, following the PRISMA guidelines for reporting findings. Finally, the review specifically focuses on the effects of auricular acupressure and its combination with other interventions.

## Conclusion

Auricular acupressure can be beneficial for insomnia. The results of our systematic review and meta-analysis of current clinical studies have shown that auricular acupressure may be as effective as estazolam in the treatment of insomnia, and it can also be used in conjunction with estazolam or other measures to improve insomnia. However, given the inclusion of low-quality studies in our analysis, our results should be interpreted with caution. In future research, it is crucial to include more high-quality studies to enhance the reliability of the conclusions drawn in this study.

## Data availability statement

The original contributions presented in the study are included in the article/Supplementary material, further inquiries can be directed to the corresponding authors.

## Author contributions

LJ: Writing – original draft, Validation, Software. LX: Writing – review & editing, Validation, Supervision, Formal analysis, Conceptualization. YW: Writing – review & editing, Project administration, Investigation, Data curation. WY: Writing – review & editing, Writing – original draft, Visualization, Investigation, Formal analysis, Data curation.
