# Supplementary material for: Effectiveness of applying auricular acupressure to treat insomnia: a systematic review and meta-analysis
Source: Front Sleep. 2024 Apr 11;3:1323967. doi: 10.3389/frsle.2024.1323967 (PMC12713953; doi:10.3389/frsle.2024.1323967)
Supplement: Supplementary file 1 [file Table_1.DOCX]

| Study | Year | Csample | Tsample | disaese | intervention | control |
| --- | --- | --- | --- | --- | --- | --- |
| Liu Liuyan | 2018 | 60 | 60 | insomnia with hypertension | auricular acupressure+usual care | usual care |
| Wei Hu | 2010 | 31 | 32 | primary insomnia | auricular acupressure | diazepam |
| Zhou Min | 2013 | 30 | 30 | insomnia with uremia | auricular acupressure | lunesta |
| Zhong Yunchun | 2019 | 20 | 20 | insomnia with uremia | auricular acupressure | usual care |
| Wu Xiuqing | 2013 | 33 | 52 | insomnia with uremia | auricular acupressure | usual care |
| Yin Chunyue | 2015 | 35 | 33 | insomnia with stroke | auricular acupressure+usual care | usual care |
| Dajun Yang | 2020 | 30 | 30 | insomnia with cancer | auricular acupressure+usual care | usual care |
| Bolan Lin | 2007 | 30 | 30 | primary insomnia | auricular acupressure | sham |
| Liu Rui | 2014 | 30 | 30 | insomnia with depression | auricular acupressure+deanxit | deanxit |
| Ren Juhong | 2015 | 38 | 34 | primary insomnia | auricular acupressure | body acupoints |
| Bao Xiuping | 2019 | 52 | 52 | insomnia with hypertension | auricular acupressure+usual care | usual care |
| Li Aiyun | 2019 | 40 | 40 | insomnia with hypertension | auricular acupressure+usual care | usual care |
| Lin Nijing | 2018 | 25 | 25 | insomnia with hypertension | auricular acupressure+usual care | usual care |
| Wang Bo | 2016 | 30 | 30 | insomnia with hypertension | auricular acupressure+estazolam | estazolam |
| Yu liyue | 2012 | 30 | 30 | insomnia with diabete | auricular acupressure | usual care |
| Chen Lu | 2018 | 40 | 40 | insomnia with AIDS | auricular acupressure | sham |
| Fan Wei | 2019 | 30 | 30 | insomnia with uremia | auricular acupressure | estazolam |
| Tan Limei | 2021 | 94 | 102 | insomnia with hypertension | auricular acupressure+anti-hypertensive | anti-hypertensive |
| Yang Guigui | 2014 | 30 | 30 | primary insomnia | auricular acupressure+estazolam | estazolam |
| Yi Pingting | 2019 | 30 | 30 | insomnia with cancer | auricular acupressure+usual care | usual care |
| Tang Aixin | 2012 | 28 | 28 | insomnia with coronary (heart) disease | auricular acupressure+usual care | usual care |
| Wang Zihao | 2020 | 25 | 25 | insomnia with stroke | auricular acupressure | diazepam |
| Sun Lihua | 2013 | 43 | 42 | insomnia with stroke | auricular acupressure | estazolam |
