# Supplementary material for: Effectiveness of applying auricular acupressure to treat insomnia: a systematic review and meta-analysis
Source: Front Sleep. 2024 Apr 11;3:1323967. doi: 10.3389/frsle.2024.1323967 (PMC12713953; doi:10.3389/frsle.2024.1323967)
Supplement: Supplementary file 2 [file Table_2.DOCX]

| Var1 | Freq |
| --- | --- |
| sympathetic(AH6a) | 14 |
| bladder(CO9) | 1 |
| large intestine(CO7) | 1 |
| pancreas and gallbladder(CO11) | 6 |
| forehead(AT1) | 1 |
| tip of ear(HX6,7i) | 2 |
| liver (CO12) | 14 |
| depressor point | 3 |
| endocrine(CO18) | 10 |
| cortex(AT4) | 13 |
| spleen (CO13) | 15 |
| sanjiao (CO17) | 3 |
| shenmen(TF4) | 22 |
| kidney(CO10) | 11 |
| stomach(CO4) | 3 |
| small intestine(CO6) | 2 |
| heart (CO15) | 19 |
| occiput (AT3) | 5 |
