# Supplementary material for: Effectiveness of applying auricular acupressure to treat insomnia: a systematic review and meta-analysis
Source: Front Sleep. 2024 Apr 11;3:1323967. doi: 10.3389/frsle.2024.1323967 (PMC12713953; doi:10.3389/frsle.2024.1323967)
Supplement: Supplementary file 3 [file Table_3.DOCX]

| type | point | number |
| --- | --- | --- |
| primary insomnia patients | sympathetic(AH6a) | 3 |
| primary insomnia patients | heart (CO15) | 4 |
| primary insomnia patients | shenmen(TF4) | 5 |
| primary insomnia patients | depressor point | 1 |
| primary insomnia patients | spleen (CO13) | 4 |
| primary insomnia patients | endocrine(CO18) | 2 |
| primary insomnia patients | liver (CO12) | 2 |
| primary insomnia patients | kidney(CO10) | 1 |
| primary insomnia patients | large intestine(CO7) | 1 |
| primary insomnia patients | small intestine(CO6) | 1 |
| primary insomnia patients | sanjiao (CO17) | 1 |
| primary insomnia patients | occiput (AT3) | 2 |
| primary insomnia patients | stomach(CO4) | 1 |
| primary insomnia patients | forehead(AT1) | 1 |
| primary insomnia patients | cortex(AT4) | 1 |
| primary insomnia patients | liver (CO12) | 2 |
| insomnia patients with uremia | shenmen(TF4) | 5 |
| insomnia patients with uremia | heart (CO15) | 5 |
| insomnia patients with uremia | sympathetic(AH6a) | 4 |
| insomnia patients with uremia | cortex(AT4) | 5 |
| insomnia patients with uremia | liver (CO12) | 2 |
| insomnia patients with uremia | tip of ear(HX6,7i) | 1 |
| insomnia patients with uremia | spleen (CO13) | 2 |
| insomnia patients with uremia | endocrine(CO18) | 3 |
| insomnia patients with uremia | kidney(CO10) | 3 |
| insomnia patients with uremia | bladder(CO9) | 1 |
| insomnia patients with uremia | spleen (CO13) | 1 |
| insomnia patients with uremia | small intestine(CO6) | 1 |
| insomnia patients with uremia | pancreas and gallbladder(CO11) | 1 |
| insomnia patients with uremia | sanjiao (CO17) | 1 |
| insomnia patients with uremia | occiput (AT3) | 3 |
| insomnia patients with stroke | shenmen(TF4) | 3 |
| insomnia patients with stroke | heart (CO15) | 3 |
| insomnia patients with stroke | liver (CO12) | 2 |
| insomnia patients with stroke | spleen (CO13) | 2 |
| insomnia patients with stroke | stomach(CO4) | 1 |
| insomnia patients with stroke | pancreas and gallbladder(CO11) | 1 |
| insomnia patients with stroke | sympathetic(AH6a) | 1 |
| insomnia patients with stroke | kidney(CO10) | 2 |
| insomnia patients with stroke | sympathetic(AH6a) | 2 |
| insomnia patients with stroke | cortex(AT4) | 3 |
| insomnia patients with stroke | endocrine(CO18) | 3 |
| insomnia patients with cancer | shenmen(TF4) | 2 |
| insomnia patients with cancer | endocrine(CO18) | 2 |
| insomnia patients with cancer | liver (CO12) | 1 |
| insomnia patients with cancer | cortex(AT4) | 1 |
| insomnia patients with cancer | spleen (CO13) | 1 |
| insomnia patients with cancer | heart (CO15) | 1 |
| insomnia patients with cancer | kidney(CO10) | 1 |
| insomnia patients with depression | shenmen(TF4) | 1 |
| insomnia patients with depression | cortex(AT4) | 1 |
| insomnia patients with depression | sympathetic(AH6a) | 1 |
| insomnia patients with depression | liver (CO12) | 1 |
| insomnia patients with depression | heart (CO15) | 1 |
| insomnia patients with depression | spleen (CO13) | 1 |
| insomnia patients with hypertension | heart (CO15) | 3 |
| insomnia patients with hypertension | liver (CO12) | 4 |
| insomnia patients with hypertension | spleen (CO13) | 3 |
| insomnia patients with hypertension | stomach(CO4) | 1 |
| insomnia patients with hypertension | kidney(CO10) | 4 |
| insomnia patients with hypertension | shenmen(TF4) | 4 |
| insomnia patients with hypertension | cortex(AT4) | 2 |
| insomnia patients with hypertension | sympathetic(AH6a) | 2 |
| insomnia patients with hypertension | tip of ear(HX6,7i) | 1 |
| insomnia patients with hypertension | sanjiao (CO17) | 1 |
| insomnia patients with hypertension | depressor point | 3 |
| insomnia patients with hypertension | endocrine(CO18) | 1 |
| 2 diabetes patients with insomnia | spleen (CO13) | 1 |
| 2 diabetes patients with insomnia | pancreas and gallbladder(CO11) | 1 |
| 2 diabetes patients with insomnia | shenmen(TF4) | 1 |
| 2 diabetes patients with insomnia | heart (CO15) | 1 |
| coronary heart patient with insomnia | shenmen(TF4) | 1 |
| coronary heart patient with insomnia | sympathetic(AH6a) | 1 |
| coronary heart patient with insomnia | cortex(AT4) | 1 |
| coronary heart patient with insomnia | heart (CO15) | 1 |
| coronary heart patient with insomnia | spleen (CO13) | 1 |
| coronary heart patient with insomnia | stomach(CO4) | 1 |
| liver depression transforming into fire | liver (CO12) | 5 |
| liver depression transforming into fire | heart (CO15) | 1 |
| liver depression transforming into fire | tip of ear(HX6,7i) | 1 |
| liver depression transforming into fire | pancreas and gallbladder(CO11) | 2 |
| phlegm-heat disturbance | spleen (CO13) | 4 |
| phlegm-heat disturbance | stomach(CO4) | 1 |
| phlegm-heat disturbance | sanjiao (CO17) | 2 |
| phlegm-heat disturbance | endocrine(CO18) | 2 |
| yin deficiency and fire hyperactivity | liver (CO12) | 3 |
| yin deficiency and fire hyperactivity | bladder(CO9) | 1 |
| yin deficiency and fire hyperactivity | endocrine(CO18) | 1 |
| yin deficiency and fire hyperactivity | kidney(CO10) | 4 |
| deficiency of both heart and spleen | spleen (CO13) | 5 |
| deficiency of both heart and spleen | heart (CO15) | 3 |
| deficiency of both heart and spleen | liver (CO12) | 1 |
| insufficiency of heart-qi and gallbladder-qi | pancreas and gallbladder(CO11) | 1 |
| insufficiency of heart-qi and gallbladder-qi | occiput (AT3) | 1 |
| none-intersecting of heart and kidney | liver (CO12) | 1 |
| none-intersecting of heart and kidney | kidney(CO10) | 1 |
| syndrome of timidity due to deficiency of heart qi | liver (CO12) | 1 |
| syndrome of timidity due to deficiency of heart qi | pancreas and gallbladder(CO11) | 1 |
| fire disturbance heart | spleen (CO13) | 1 |
| fire disturbance heart | large intestine(CO7) | 1 |
| stagnation of qi due to depression of the liver? | liver (CO12) | 1 |
| stagnation of qi due to depression of the liver? | sanjiao (CO17) | 1 |
