# Supplementary figures and images for: Effectiveness of applying auricular acupressure to treat insomnia: a systematic review and meta-analysis
Source: Front Sleep. 2024 Apr 11;3:1323967. doi: 10.3389/frsle.2024.1323967 (PMC12713953; doi:10.3389/frsle.2024.1323967)

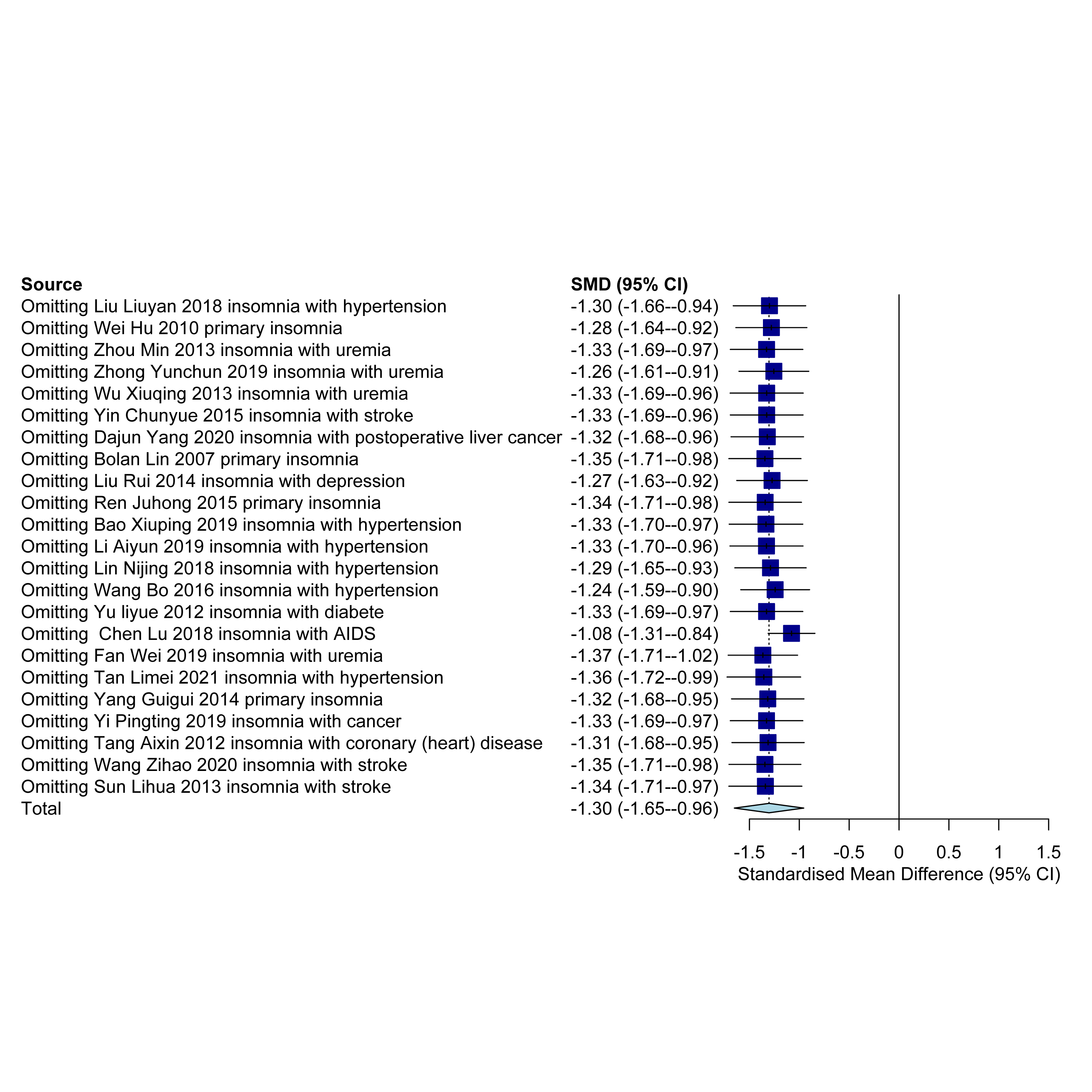

Supplement: Supplementary file 4 [file Image_1.TIF]

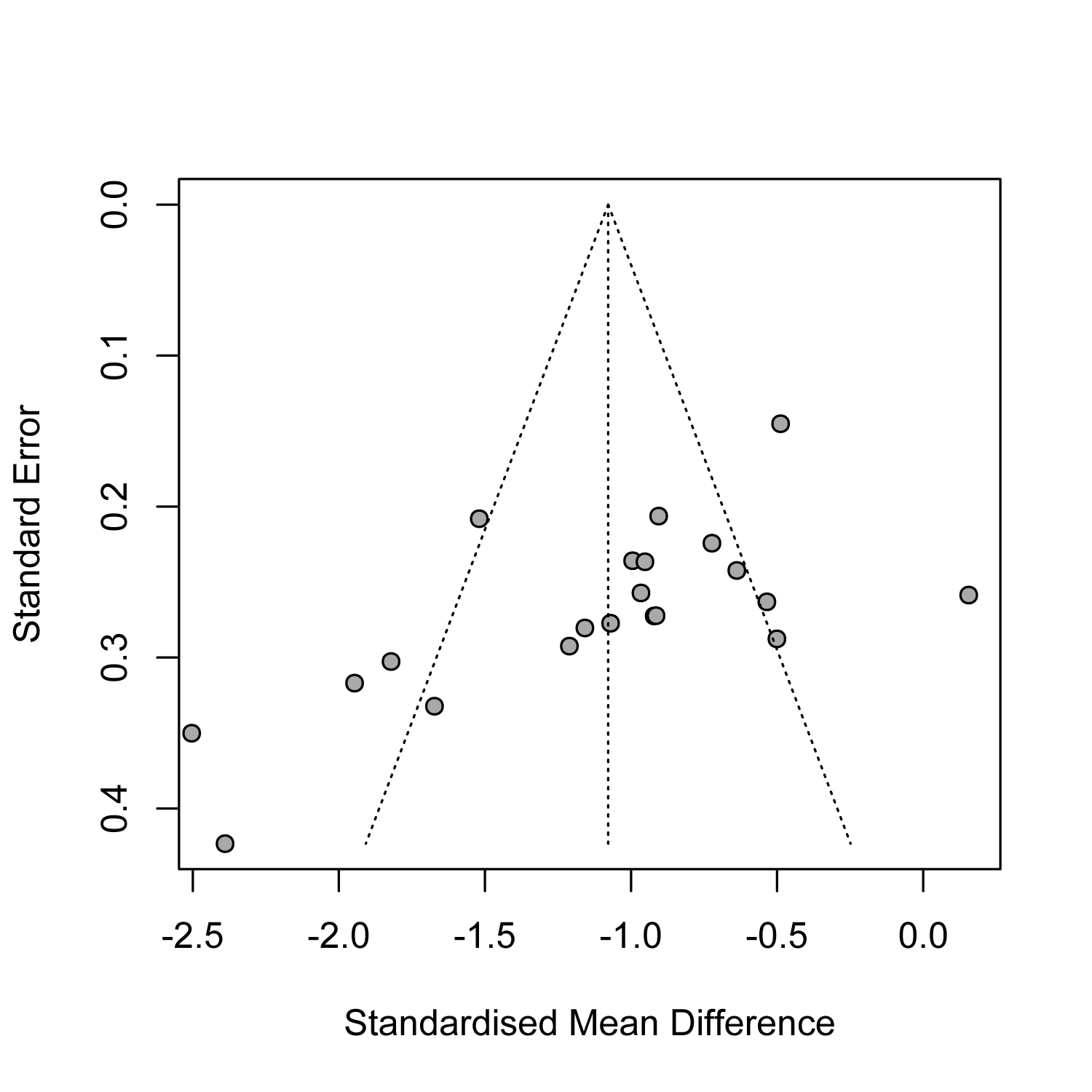

Supplement: Supplementary file 5 [file Image_2.TIF]
